# Variant in the X-chromosome spliceosomal gene *GPKOW* causes male-lethal microcephaly with intrauterine growth restriction

**DOI:** 10.1038/ejhg.2017.97

**Published:** 2017-06-14

**Authors:** Renée Carroll, Raman Kumar, Marie Shaw, Jennie Slee, Vera M Kalscheuer, Mark A Corbett, Jozef Gecz

**Affiliations:** 1 The Robinson Research Institute, The University of Adelaide, Adelaide, Australia; 2 School of Medicine, The University of Adelaide, Adelaide, Australia; 3 Genetic Services of Western Australia, King Edward Memorial Hospital, Subiaco, WA, Australia; 4 Research Group Development and Disease, Max Planck Institute for Molecular Genetics, Berlin, Germany; 5 School of Biological Sciences, The University of Adelaide, Adelaide, SA, Australia; 6 South Australian Health and Medical Research Institute, Adelaide, SA, Australia

## Abstract

Congenital microcephaly, with or without additional developmental defects, is a heterogeneous disorder resulting from impaired brain development during early fetal life. The majority of causative genetic variants identified thus far are inherited in an autosomal recessive manner and impact key cellular pathways such as mitosis, DNA damage response and repair, apoptosis and splicing. Here, we report a novel donor splice site variant in the G-patch domain and KOW motifs (*GPKOW*) gene (NG_021310.2:g.6126G>A, NM_015698.4:c.331+5G>A) that segregates with affected and carrier status in a multigenerational family with an X-linked perinatal lethal condition characterized by severe microcephaly and intrauterine growth restriction (IUGR). GPKOW is a core member of the spliceosome that has been shown in numerous model organisms and in human cells to be essential for survival. By investigating *GPKOW* transcripts in lymphoblastoid cell lines (LCLs) of three carrier females, we show that the *GPKOW* c.331+5G>A variant disrupts normal splicing of its pre-mRNAs. In a clonal culture expressing only the c.331+5G>A allele isolated from one carrier female LCL, we observed an 80% reduction in wild type *GPKOW* mRNA, 70% reduction in the full length GPKOW protein and the presence of a truncated GPKOW protein with possible dominant negative effect. Based on our and published data we propose that the *GPKOW* gene is essential for fetal development and when disrupted, leads to a severe, male-lethal phenotype characterised by microcephaly and IUGR.

## Introduction

Congenital microcephaly, a condition where head circumference at birth is significantly below the mean for age and sex, is the result of impaired brain development during early fetal life.^1^ While the genetic causes are diverse, causative variants predominantly show an autosomal recessive mode of inheritance and affect fundamental cellular pathways such as the assembly and function of the mitotic apparatus; DNA replication, damage and repair; apoptosis signaling and mRNA splicing.^1, 2^ Why the developing fetal brain is particularly sensitive to even subtle alterations in pathways core to all living cells is still being investigated.^2, 3^

We studied a previously reported multigenerational family with a male lethal condition primarily characterized by severe microcephaly with IUGR and variable presentation of additional developmental defects,^4^ with carrier females possibly presenting with a mild phenotype. We identified a rare variant in a canonical donor splice site of the X-linked splicing factor gene *GPKOW* as the most likely cause of this condition.

## Materials and methods

Details of materials and methods can be found in the Supplementary Information.

## Results

**Figure 1:**
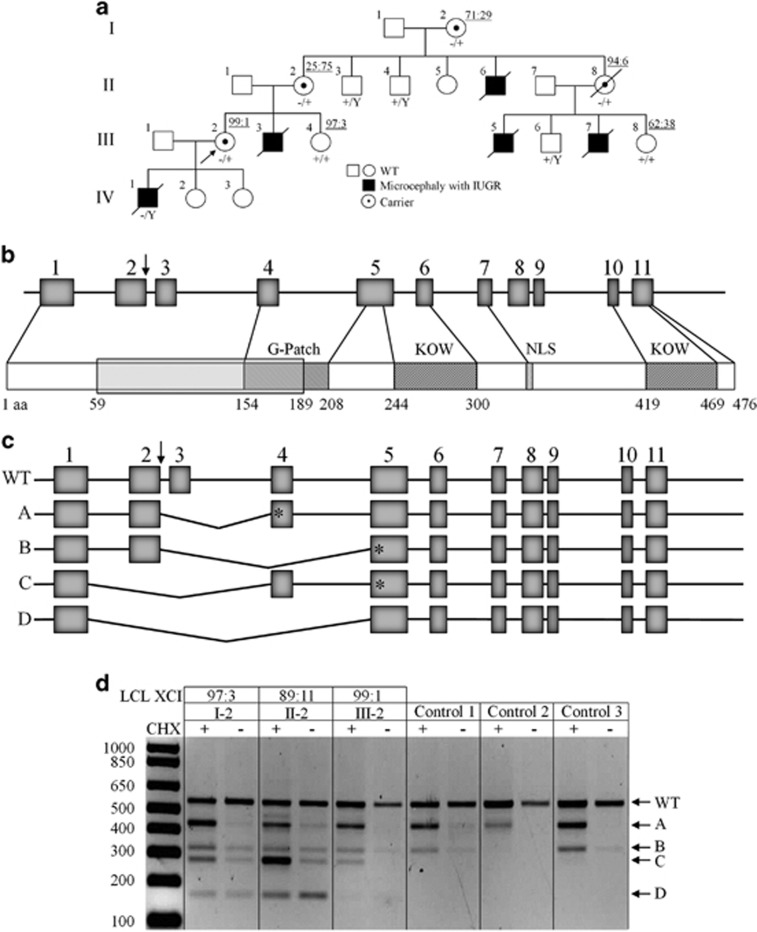
(**a**) Family pedigree. Genotyped individuals are indicated as WT +/Y or +/+ carrier −/+ affected -/Y. XCI ratios (peripheral blood DNA) for the tested females are shown (Supplementary Table 3). Foetus II-6 was a missed miscarriage at 25/40, III-3 died in the neonatal period at 36/40, III-5 was stillborn at 34/40, and pregnancies III-7 and IV-1 were terminated at 21/40 and 18.5/40, respectively, following diagnosis by ultrasound. IUGR, intrauterine growth restriction. (**b**) Schematic structure of *GPKOW* gene and protein. Arrow indicates location of +5G>A variant in the donor splice site of exon 2. One G-Patch domain, two KOW motifs and a predicted nuclear localisation sequence (NLS, aa 331–334) are shown. Exons are numbered as in NCBI Refseq NG_021310.2 and the amino acid locations of different domains are from the NCBI Refseq NP_056513.2. The shaded box indicates the aa 59–189 missing from the truncated protein, based on transcript D cDNA sequence. (**c**) Schematic representation of the four different transcripts identified in the cycloheximide (CHX) assay. Asterisks represent premature termination codons (PTC) introduced due to shifts in reading frame. (**d**) c.331+5G>A carrier female LCLs show additional aberrant processing of *GPKOW* pre-mRNA. Agarose gel showing *GPKOW* transcripts amplified from carrier and control female LCLs cultured in the presence (+) or absence (–) of CHX. Full-length (WT) and aberrantly-spliced (A–D) transcripts are indicated by arrows. XCI ratios assayed from LCL gDNA from three carrier females are also shown.

X-exome sequencing of obligate carrier III-2 (Figure 1a) and subsequent variant filtering identified five novel heterozygous variants (Supplementary Table 1), of which the *GPKOW* (NG_021310.2:g.6126G>A; NM_015698.4:c.331+5G>A) and *IRS4* (NG_013239.1:g.7189C>T; NM_003604.2:c.2156C>T) variants were considered candidates. However, only the *GPKOW* c.331+5G>A splice-site variant segregated in nine additional family members, including one affected male fetus (Figure 1a and Supplementary Table 3). The *GPKOW* c.331+5G>A did not have a damaging CADD score^5^ (Supplementary Table 1), however different *in silico* prediction tools calculated a decrease in splicing efficiency as compared to wild type sequence (Supplementary Table 2). *GPKOW* has a probability of loss-of-function intolerance Z score of 0.98 in ExAC,^6^ suggesting that disruption of this gene is likely to be damaging (Supplementary Table 1).

Extreme skewing (> 90%) of X chromosome inactivation (XCI) was observed in blood-derived genomic DNA from three females; II-8, III-2 and III-4 (Figure 1a), however skewing of XCI ratios did not co-segregate with obligate carrier status or presence of the *GPKOW* variant. Namely, I-2 and II-2 are obligate carriers and heterozygous for *GPKOW* c.331+5G>A variant yet have random XCI, whereas III-4, who has not conceived affected children and is homozygous wild type (WT) at this locus, has skewed XCI to the ratio of 97:3. Furthermore, skewed XCI, as assessed on venous blood DNA, does not appear to be fully protective against the effect of the *GPKOW* variant in carrier females. For example, II-8 has highly skewed XCI yet her height and occipitofrontal circumference (OFC) measurements are in the <1st and 2nd centiles, respectively. Incidentally, we observed a missense variant in exon 1 of the *AR* gene (NG_009014.2:g.6285T>A, NM_000044.3:c.170T>A, p.(Leu57Gln), rs78686797) that is in linkage disequilibrium with the *GPKOW* c.331+5G>A variant (Supplementary Figure 1c). This was supported by determining the *AR* allele inheritance for affected and unaffected male family members (Supplementary Table 3). Subsequently, we used the methylation status of the *AR* sequence tagged site (NCBI Probe database Pr012386469) that flanks rs78686797 as a proxy to indicate WT or variant *GPKOW* allele expression. LCLs from carrier females I-2, II-2 and III-2 were all highly skewed toward expression of the WT *GPKOW* allele (Supplementary Figure 3). XCI measured on DNA from LCLs differed to XCI measured from venous blood DNA for obligate carriers I-2 and II-2 (see Supplementary Figure 3A).

Given the severity of the disorder we did not have access to a viable cell source from an affected male. To investigate whether the *GPKOW* c.331+5G>A donor splice site variant alters *GPKOW* pre-mRNA splicing and creates alternative mRNA species harboring premature termination codons (PTC) that are subject to Nonsense Mediated mRNA Decay (NMD), we inhibited NMD in low passage LCLs from three carrier (I-2, II-2 and III-2) and three control females with cycloheximide (CHX). Increased levels of bona fide NMD target *GADD45B* mRNA confirmed NMD inhibition (Supplementary Figure 2). RT-PCR amplification of *GPKOW* mRNA using primers located within exons 1 and 5 revealed four aberrant *GPKOW* transcripts in addition to the full-length transcript (Figures 1b and d). Transcripts A and B were present in all CHX-treated LCLs suggesting they result from normal splicing events unrelated to the c.331+5G>A variant, and as they were largely undetectable in the untreated LCLs, are naturally degraded by NMD. Sanger sequencing revealed transcript A lacks exon 3 and transcript B lacks exons 3 and 4 (Figures 1c and d and Supplementary Figure 1b), in both cases causing frameshifts and the introduction of a PTC. Transcripts C and D were only amplified from carrier female samples, suggesting they result from incorrect processing of the allele with the variant c.331+5G>A donor splice site. Transcript C was predominantly detected in the CHX-treated samples indicating it is subject to NMD. Sanger sequencing revealed transcript C lacks exons 2 and 3, causing frameshift and the introduction of a PTC. Presence of comparable levels of transcript D in CHX-treated and untreated carrier female LCLs suggested that it is refractory to NMD. Sanger sequencing showed that transcript D lacks exons 2, 3 and 4, maintaining the reading frame (Figures 1c and d and Supplementary Figure 1b). The differences in the abundance of transcripts C and D observed between the carrier female LCLs (Figure 1d) corresponded to the percentage of the cells in each culture expressing the *GPKOW* c.331+5G>A allele, as determined by methylation status of the *AR* c.170T>A, p.(Leu57Gln) allele.

**Figure 2:**
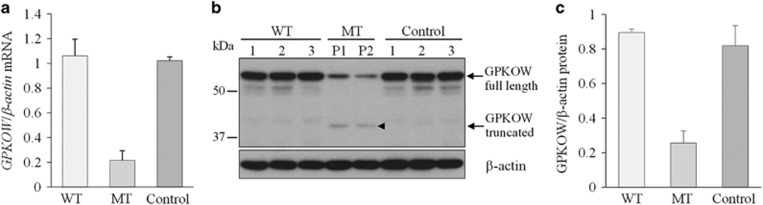
GPKOW full length WT mRNA and protein are significantly reduced in the c.331+5G>A LCL clone (MT). (**a**) Graph showing *GPKOW* mRNA levels relative to *ACTB*. RT-qPCR data from independent WT clones (*n*=3), c.331+5G>A clones (*n*=2; different passages of the same culture) from the carrier female II-2, and normal controls (*n*=3) was averaged and error bars show SD. (**b**) Western blot showing full length GPKOW from independent WT clones (*n*=3), the c.331+5G>A clone (*n*=2; different passages of the same culture; P1, P2), and normal controls (*n*=3). An additional band at around 40kDa in the c.331+5G>A (MT) clones P1 and P2 corresponds to the predicted size of a truncated protein translated from transcript D. (**c**) Graph showing the mean densitometry values of full length GPKOW protein relative to β-actin. Western blot signals in (**b**) were measured by ImageJ software and normalised to housekeeping β-actin protein. Error bars show SD.

**Figure 3:**
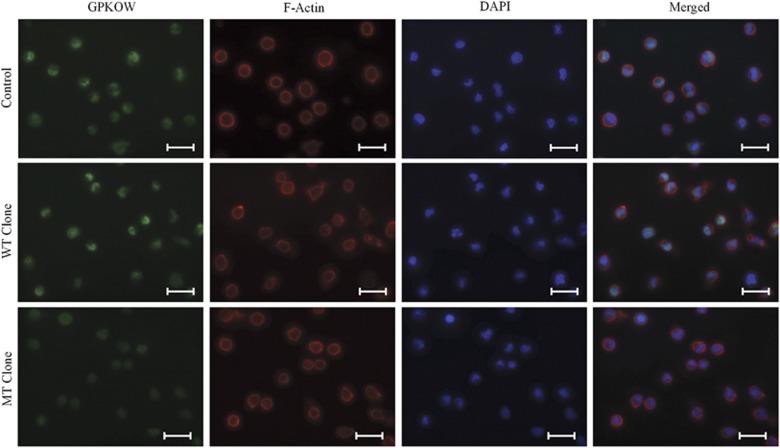
Immunofluorescence showing a dramatic GPKOW reduction in c.331+5G>A clonal LCLs. Representative images of LCL immunofluorescence for GPKOW from normal controls from different individuals (*n*=3), independent WT clones (*n*=3) and c.331+5G>A clone (*n*=1, done in duplicate). Scale bars represent 20 μm.

To further investigate the c.331+5G>A variant in isolation from sequence-confirmed WT allele expression, we isolated clonal cultures from the LCL of carrier female II-2. From 13 successfully isolated clones, 12 expressed the WT *GPKOW* allele and one the c.331+5G>A variant allele (Supplementary Figure 3b). Real time quantitative PCR (RT-qPCR) revealed that full length *GPKOW* levels were reduced by 80% in the c.331+5G>A expressing clone as compared to three independent WT expressing clones and three unrelated controls (Figure 2a). Western blot analysis showed a corresponding 70% reduction in full-length GPKOW protein, with the presence of an additional band at approximately 40 kDa, corresponding to the size predicted for transcript D translation product (Figures 2b and c). Indirect immunofluorescent detection of GPKOW protein in clonal LCL cultures demonstrated significant reduction in GPKOW levels but no discernible effect on its localization in the c.331+5G>A variant cells compared to WT cells and unrelated controls (Figure 3).

## Discussion

That RNA metabolism is fundamental to normal brain development and neuronal function is demonstrated by the complexity of alternative precursor messenger RNA (pre-mRNA) splicing in the human brain.^7^ In excess of 350 disorders, including many neurodevelopmental disorders, are consequences of genetic variants affecting pre-mRNA splicing.^8^ Furthermore, variants in splicing factors themselves are reported to cause a wide variety of human diseases.^8, 9, 10^

In this work we implicate *GPKOW* in a male-lethal developmental disorder. GPKOW is a ubiquitously expressed nuclear RNA-binding protein that has been identified as a core member of the spliceosome.^11^ GPKOW associates with catalytically active complexes B^act^ and C^11, 12^ and acts as an essential cofactor for the two DEAH-box helicases, DHX16 and DHX38, which sequentially catalyze the key transesterification reactions. *GPKOW* has been classified as essential for human cell survival,^13^ and there are no known paralogs in the human genome. Moreover, there are no homozygous or hemizygous loss of function variants thus far listed in ExAC.^6^ However, DECIPHER lists a patient (#286121) with a 1.4 Mbp deletion including the *GPKOW* gene and a 33 Mbp pathogenic deletion of chromosome 21, presenting microcephaly as one of the clinical symptoms. *GPKOW* is highly conserved and has been shown to be essential for life in numerous model organisms. For example, in *Caenorhabditis elegans*, RNAi manipulation of its *GPKOW* ortholog R11A8.2 results in embryonic lethality in more than 80% of cases and the minority that survived had morphological growth defects.^14^ RNAi silencing of the *GPKOW* ortholog CG10324 in *Drosophila melanogaster* neuroblasts is early lethal and causes under proliferation.^15^ To our knowledge, no vertebrate model of a *GPKOW* knockout has been generated.

The *GPKOW* c.331+5G>A variant we identified was predicted *in silico* to adversely impact spliceosomal processing of exon 2 donor splice site (Supplementary Table 2). Although adenine at the +5 position occurs in approximately 9% of human donor splice sites genome-wide,^16^ there are numerous reported cases of +5G>A disease-associated variants causing aberrant pre-mRNA processing.^17^ Blocking NMD in LCLs from three carrier females revealed the expression of the c.331+5G>A allele is correlated with the production of aberrant transcripts C and D. Current evidence shows that *GPKOW* is subject to XCI^18^ and as such in a female the gene is expressed only from the active X-chromosome. Using clonal WT and c.331+5G>A variant expressing LCL cultures from one carrier female we show the likely effect of the c.331+5G>A variant on *GPKOW* mRNA splicing. In the c.331+5G>A allele-expressing LCL clone, we observed full-length transcript levels were reduced by 80% as compared to expression levels in WT-expressing cultures (Figure 2a). Importantly, we also observed a corresponding 70% reduction in full-length GPKOW protein, and the presence of a truncated GPKOW protein, corresponding to the transcript D translation product (Figures 2b and c). The truncated protein is predicted to lack amino acids 59–189 (Figures 1b and c), spanning the first two-thirds of the G-patch domain, which has been shown to be important for GPKOW binding to DHX16.^12^ Consequently, we predict that the truncated protein, even if folded and localized correctly, would be unlikely to function properly as a cofactor in spliceosomal reactions. We also speculate that the truncated GPKOW protein may have a dominant negative effect by competing with residual WT GPKOW for binding sites and/or binding to and sequestering other spliceosomal factors.

Testing of X-chromosome inactivation provided conflicting data. Three females (II-8, III-2 and III-4) of whom two are obligate carriers (II-8 and III-2), had highly skewed XCI, while two other obligate carriers (I-2 and II-2) showed random XCI. However, when we tested XCI on DNA isolated from LCLs of three obligate carriers, two of whom have variable XCI in blood (I-2 and II-2), we found all three to have highly skewed XCI favoring expression of the wild type *GPKOW* allele (Supplementary Figure 3a). Skewing of XCI also doesn’t appear to be protective of the possible mild phenotype observed in the obligate carrier females (Supplementary Table 3). On aggregate, the combined data on XCI in blood and LCL DNA and on multiple obligate carrier and wild type females in this family show a complex, yet to be resolved mechanism of XCI skewing in different tissues. It also appears that a factor unrelated to the *GPKOW* variant and involved in high XCI skewing, might also be segregating in this family (e.g. wild type female III-4 with high skewing of XCI; Figure 1a).

How significantly reduced levels of WT GPKOW along with any function of the truncated GPKOW lead to the observed male-lethal phenotype remains to be determined. However, there are a number of severe congenital disorders with the shared features of microcephaly and growth retardation that are attributed to genetic variants in splicing factors. For example, mandibulofacial dysostosis, Guion-Almeida type (MFDGA, OMIM #610536) is caused by heterozygous variants in *EFTUD2*; Verheij syndrome (OMIM #615583) has recently been attributed to heterozygous variants in *PUF60*;^19^ and microcephalic osteodysplastic primordial dwarfism type I (MOPD1, OMIM #210710) caused by recessive variants in *RNU4ATAC*. Furthermore, the particular sensitivity of males to mutations in X-linked splicing factors is demonstrated with the example TARP syndrome (OMIM #311900), a male-lethal developmental disorder caused by mutations in the X-linked gene RNA Binding Motif Protein 10 (*RBM10*).^20^

Our findings show that a variant in the X-chromosome essential splicing factor GPKOW is the likely cause of a male-lethal disorder featuring microcephaly and IUGR, and add to the growing number spliceosomal gene variants implicated in severe developmental disorders, highlighting the crucial role of pre-mRNA splicing in human development.^8^ Further investigations to elucidate the role of GPKOW in normal development are warranted.
