# Supplementary material for: Variant in the X-chromosome spliceosomal gene GPKOW causes male-lethal microcephaly with intrauterine growth restriction
Source: Eur J Hum Genet. 2017 Jun 14;25(9):1078–82. doi: 10.1038/ejhg.2017.97 (PMC5558180; doi:10.1038/ejhg.2017.97)
Supplement: Supplementary File [file ejhg201797x1.docx]

**SUPPLEMENTAL METHODS, FIGURES AND TABLES**

**Title: Variant in the X-chromosome spliceosomal gene *GPKOW* causes male-lethal microcephaly with intrauterine growth restriction**

**Running title: *GPKOW* variant in microcephaly with IUGR**

Renée Carroll^1,2,#^ , Raman Kumar^1,2,#^, Marie Shaw^1,2^, Jennie Slee^3^, Vera M. Kalscheuer^4^, Mark A. Corbett^1,2^, Jozef Gecz^1,2,5,6*^

^1^The Robinson Research Institute, The University of Adelaide, Adelaide, SA 5000, Australia, ^2^School of Medicine, The University of Adelaide, Adelaide, SA 5000, Australia, ^3^Genetic Services of Western Australia, King Edward Memorial Hospital, Subiaco, WA 6008, Australia, ^4^Research Group Development and Disease, Max Planck Institute for Molecular Genetics, Berlin, 14195, Germany, ^5^School of Biological Sciences, The University of Adelaide, Adelaide, SA 5000, Australia, ^6^South Australian Health and Medical Research Institute, Adelaide, SA 5000, Australia

^#^These authors contributed equally to this work.

^*^To whom correspondence should be addressed: School of Medicine and The Robinson Research Institute, The University of Adelaide, Adelaide, SA 5000, Australia. Tel: +61 883133245; Fax: +61 881617342; Email: [jozef.gecz@adelaide.edu.au](mailto:jozef.gecz@adelaide.edu.au)

**METHODS**

**Ethical statement**

The study was approved by the Women’s and Children’s Health Network Human Research Ethics Committee and written informed consent was obtained from the participating family.

**Clinical report**

Individual II-2 (Figure 1a) was referred to Genetic Services in 1981 following the death at 2 minutes post-delivery of a male at 36 weeks of gestation (III-3: Figure 1a) with growth retardation, microcephaly and talipes equinovarus. Birth weight was 838 g. An ultrasound at 30 weeks revealed IUGR and microcephaly. Post mortem examination revealed marked hydroencephaly with just a thin mantle of cortical tissue, hypoplastic lungs and very small but mature kidneys.

Individual II-8 (Figure 1a) was referred following the delivery of a growth restricted stillborn microcephalic male (III-5: Figure 1a) at 34½ weeks of gestation, weighing 1300 g. Ultrasound scans 6 days earlier revealed extreme microcephaly with biparietal diameter (BPD) of 41 mm (equivalent to 17 weeks of gestation) in association with hydrops and ‘plentiful’ liquor. Earlier scan at 19 weeks showed a small BPD of 31 mm (equivalent to 15 weeks of gestation). Post mortem examination revealed extreme microcephaly, hypoplastic lungs and small but normally formed kidneys. A small amount of neural tissue could be identified adherent to the skull. There was loss (presumed regression) of the telencephalon, prosencephalon and diencephalon with subsequent hydroanencephaly. This male also had penile chordae tendinae.

In 1990, in another pregnancy (III-7: Figure 1a), serial ultrasound examinations commencing at 18 weeks of gestation showed marked microcephaly and possible holoprosencephaly. The pregnancy was terminated at 21 weeks of gestation with a birth weight of 150g. There was obvious microcephaly with head circumference of 12.3 cm and a sloping forehead and small eyes. Microscopy of the brain revealed cortical gyral development of around 15 weeks of gestation. The optic nerves and optic chiasm could not be visualised. The feet had a rocker bottom appearance with profound lateral deviation.

Individual III-2 (Figure 1a) was referred early in her first pregnancy to discuss the family history and arrange serial ultrasound scans. At 15 weeks of gestation the gender was determined to be male (IV-1: Figure 1a) and there was already evident microcephaly (BPD 22 mm) and ventriculomegaly, together with growth restriction. At 18 weeks the BPD measured at 29 mm and the degree of ventriculomegaly had increased. The amniotic fluid, which had been normal at 15 weeks, was increased at 18 weeks of gestation. The birth weight was just 89.5 g at 18½ weeks of gestation at delivery. Autopsy examination showed small but normally formed lungs and kidneys. The eyes were small and the orbital contents consisted of predominantly loose connective tissue with a small amount of very immature retinal type epithelium. The CNS showed two separate cerebral hemispheres, no clear corpus callosum and an apparent doubling of the cortex laterally, suggesting a neuronal migration defect.

Individuals II-2 and II-8 (Figure 1a) were both of short stature with small head circumferences. Individual II-2 was of height 150.6 cm (3rd percentile) with head circumference 51.8 cm (below the 2nd centile) and Individual II-8 was of height 144 cm (below the 1st percentile) with head circumference 52.5 cm (on the 2nd centile). The unaffected males (II-3 and II-4: Figure 1a) were of normal stature and head circumference. Both males were 182 cm tall (75th centile) and with head circumference 58.5 cm (75th percentile). Individual III-2 was on the 50th centile for height (162.5 cm) and between the 2nd and 10th centiles for head circumference (53.2 cm).

**X-exome sequencing and analysis**

DNA from obligate carrier female III-2 (Figure 1a) was extracted from blood (QIAamp DNA blood maxi kit; Qiagen, Hilden, Germany) and X-chromosome exome sequencing was performed as part of a large sequencing study^1^. Given the lethal phenotype, identified variants were disregarded if they were present in hemizygotes in gnomAD^2^. Novel variants were considered further if they were predicted to be deleterious by a combination of *in silico* predictions^1^ and functional evidence from molecular studies and model organisms (Supplementary Tables 1 and 2). Variant submitted to Leiden Open Variation Database version 3.0 (http://databases.lovd.nl/shared/individuals/00102741).

**Sanger sequencing**

Confirmation of variants and segregation analysis was performed by Sanger sequencing using standard methods. Primer sequences including those used for sequencing the open reading frame, 5’ and 3’ UTR of *GPKOW* in carrier females, are listed in Supplementary Table 4.

**Cell culture**

LCLs were established for three obligate carrier females I-2, II-2 and III-2. Peripheral blood lymphocytes were isolated and transformed with Epstein Barr Virus using standard protocols^3^. Established cultures were grown in RPMI-1640 medium (Sigma-Aldrich) supplemented with 10% FBS (Gibco; Thermo Fisher Scientific, Waltham, MA, USA), 2 mM L-glutamine (Sigma-Aldrich), and 0.15 mg/ml benzylpenicillin (CSL Limited, Melbourne, Australia). Prior to any subdivisions, a single cell suspension was performed in order to homogenize the cultures and reduce the risk of mechanically skewing the XCI ratios.

**XCI analysis of blood and LCL genomic DNA**

XCI ratios were determined for peripheral blood and LCL cultures using the *FRAXA* and *AR* polymorphic markers as described previously^4,5^ (primer sequences used for XCI are listed in Supplementary Table 4). To test genetic linkage to the *AR* locus, *AR* allele inheritance was determined on undigested gDNA for affected and unaffected male family members for whom gDNA was available, using fragment analysis and Sanger sequencing.

**Cycloheximide assay, RT-qPCR and *GPKOW* transcript analysis**

Approximately 3×10^6^ cells from early passage LCL cultures from three carrier and three unrelated control females were incubated with either 100 μg/ml cycloheximide (Sigma-Aldrich) or 0.01% DMSO (Sigma-Aldrich) at 37°C with 5% CO_2_ for 6 hours. Cells were then harvested, washed once in 1×PBS (Sigma-Aldrich), and snap frozen until RNA extraction using RNeasy Mini Kit (Qiagen) and generation of cDNA as per standard methods. An additional pellet of untreated cells from each individual was simultaneously harvested and genomic DNA extracted using DNeasy Blood and Tissue kit (Qiagen) for XCI analysis. Successful inhibition of NMD was confirmed by RT-qPCR for a bona fide NMD target *GADD45B* normalized to *HPRT1* using SYBR Green for detection of products and relative standard curve for quantification (Bio-Rad Laboratories, Hercules, CA, USA). Primers are listed in Supplementary Table 4. The presence of different *GPKOW* transcripts was determined by PCR amplification performed on cDNA from CHX-treated and -untreated LCLs as per standard methods with *GPKOW* primers located in exons 1 and 5 (Supplementary Table 4). Amplified products were resolved on a 1.5% agarose gel. Specific DNA bands were purified by gel extraction (QIAquick Gel Extraction Kit, Qiagen), and sequenced by Sanger sequencing.

**Isolation of LCL clones and *GPKOW* mRNA and protein assays**

Clonal LCL cultures expressing only the WT or c.331+5G>A *GPKOW* alleles were isolated from carrier female II-2 LCL by culturing the cells in 96-well plates at a density of approximately 1 cell per well. Resulting cultures were expanded and gDNA was extracted (DNeasy Blood and Tissue kit; Qiagen) to determine their XCI status as proxy for specific *GPKOW* allele expression. mRNA from selected clonal (WT and c.331+5G>A expressing cultures) and unrelated normal control LCLs were reverse transcribed and used for qPCR with primers bridging exon junctions 2-3 and 3-4 to specifically determine full-length *GPKOW* transcript levels. Protein lysates were prepared in lysis buffer (50 mm Tris-HCl, pH 7.4, 0.1% Triton X-100, 5 mM EDTA, 250 mM NaCl, 1× protease and phosphatase inhibitor cocktails, Roche Applied Sciences) followed by sonication and centrifugation. Lysates were resolved by SDS-PAGE on a 12% gel and transferred onto nitrocellulose membrane (Amersham Biosciences). Blot was probed for GPKOW (0.2 μg/ml; HPA-001894, Sigma), detected with anti-rabbit horseradish peroxidase-conjugated secondary antibody (Dako) and visualized by enhanced chemiluminescence (GE Healthcare). Blot was stripped using mild stripping buffer (Abcam standard protocols) and re-probed for β-actin loading control (Sigma).

**LCL immunofluorescence**

Approximately 10^5^ LCLs were plated onto BD BioCoat poly-L-lysine coverslips and processed using a novel protocol to be published separately. Primary GPKOW (0.4 μg/ml; HPA-001894, Sigma) or rabbit IgG (Sigma), and secondary Alexa Fluor 488 donkey anti-rabbit (A-21206, Thermo Fisher, used at 1/2500 dilution) antibodies were used. Filamentous actin was stained using Phalloidin TRITC (Sigma), and slides were mounted using ProLong Gold Antifade Mountant with DAPI (Thermofisher). Images were captured with an AxioVision camera on a Carl Zeiss microscope.

**SUPPLEMENTAL FIGURES**

**Supplementary Figure 1** Sequencing chromatograms. (**a**) Segregation analysis of the *GPKOW* c.331+5G>A variant. (**b**) Four *GPKOW* alternative transcripts identified in CHX assay. (**c**) The *AR* c.170T>A p.(Leu57Gln) variant (arrowed) and how this allele appears in fragment analysis.


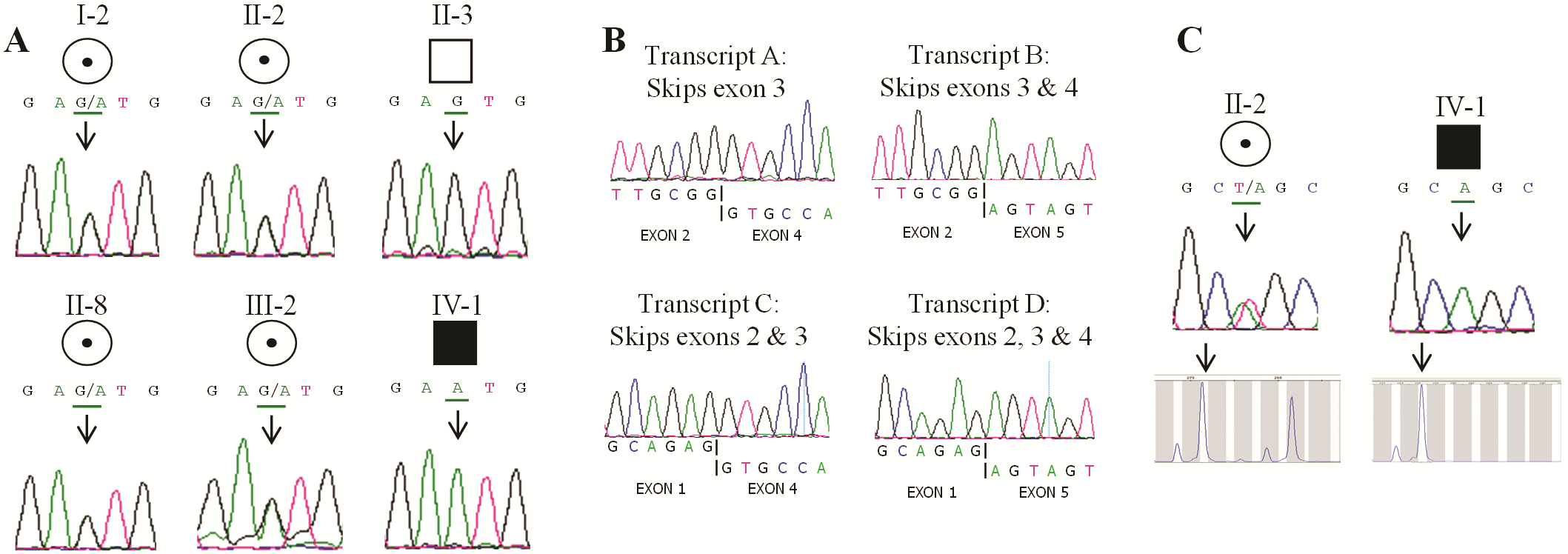


**Supplementary Figure 2** NMD was successfully inhibited in CHX treated LCLs. Levels of the known endogenous NMD target *GADD45B* mRNA relative to *HPRT1* are significantly higher in CHX treated (+) versus untreated (-) samples showing successful inhibition of NMD.


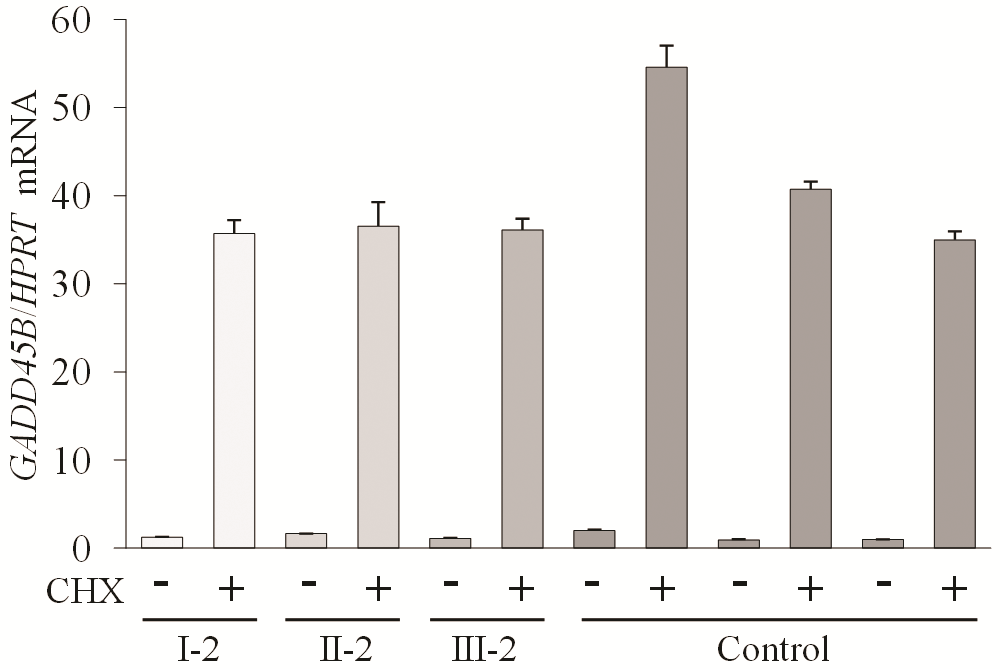


**Supplementary
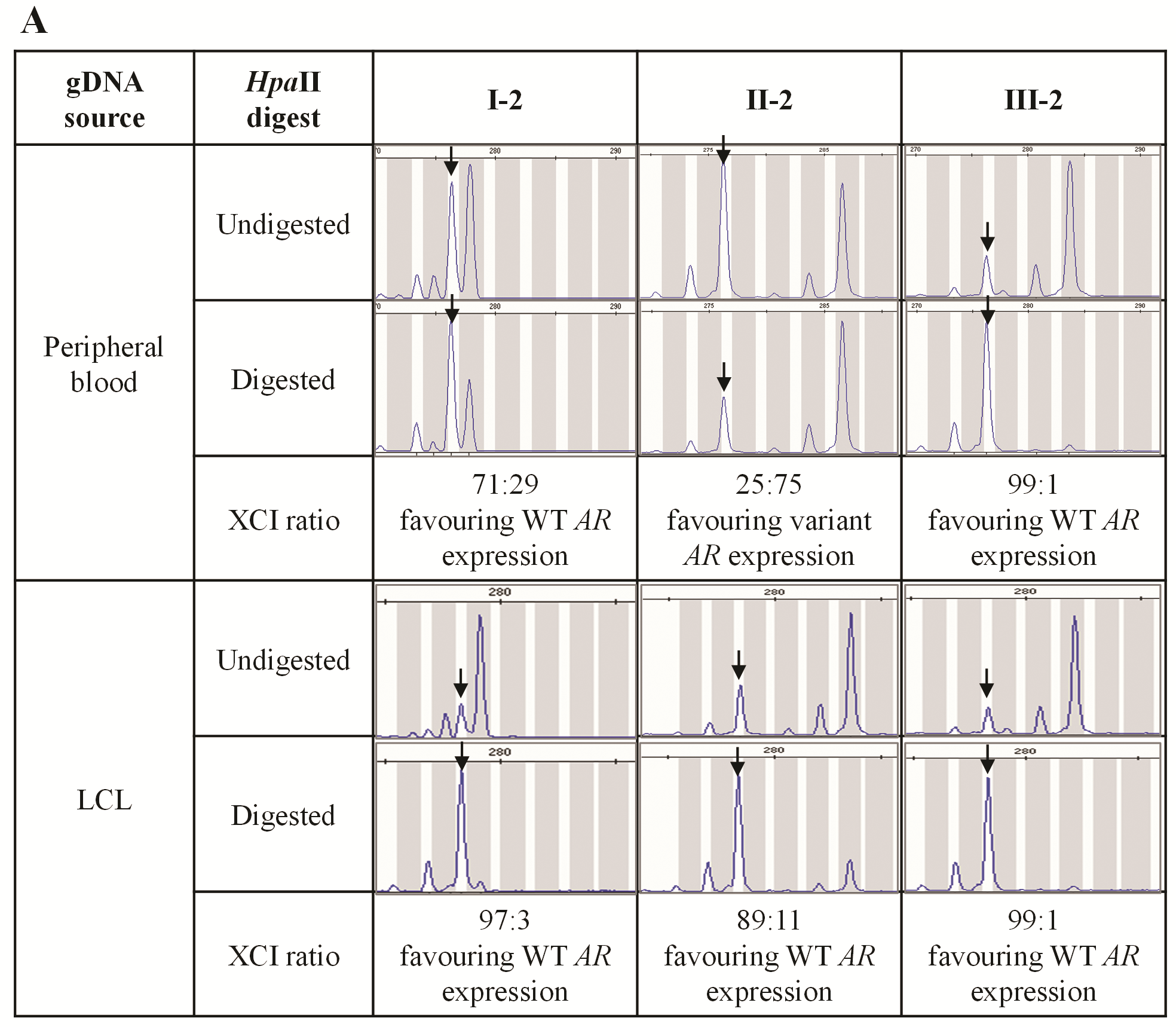
 Figure 3** XCI for *AR* shows the variant allele in linkage with the *GPKOW* variant is preferentially inactivated in LCLs, irrespective of peripheral blood XCI results. (**a**) Fragment analysis of *AR* alleles amplified from *Hpa*II digested and undigested gDNA from peripheral blood and LCL cultures for carrier females I-2, II-2 and III-2, with corresponding XCI ratios. Arrows point to the unique peak corresponding to the *AR* c.170T>A p.(Leu57Gln) variant allele that is in linkage disequilibrium with the *GPKOW* c.331+5G>A variant allele. (**b**) Fragment analysis of *AR* alleles from the 3 independent WT and 1 MT clonal cultures isolated from carrier II-2 LCL and used in experiments. WT or MT expression status for the GPKOW c.331+5G>A variant is determined by inactivation or expression of the linked *AR* c.170T>A p.(Leu57Gln) variant, respectively.


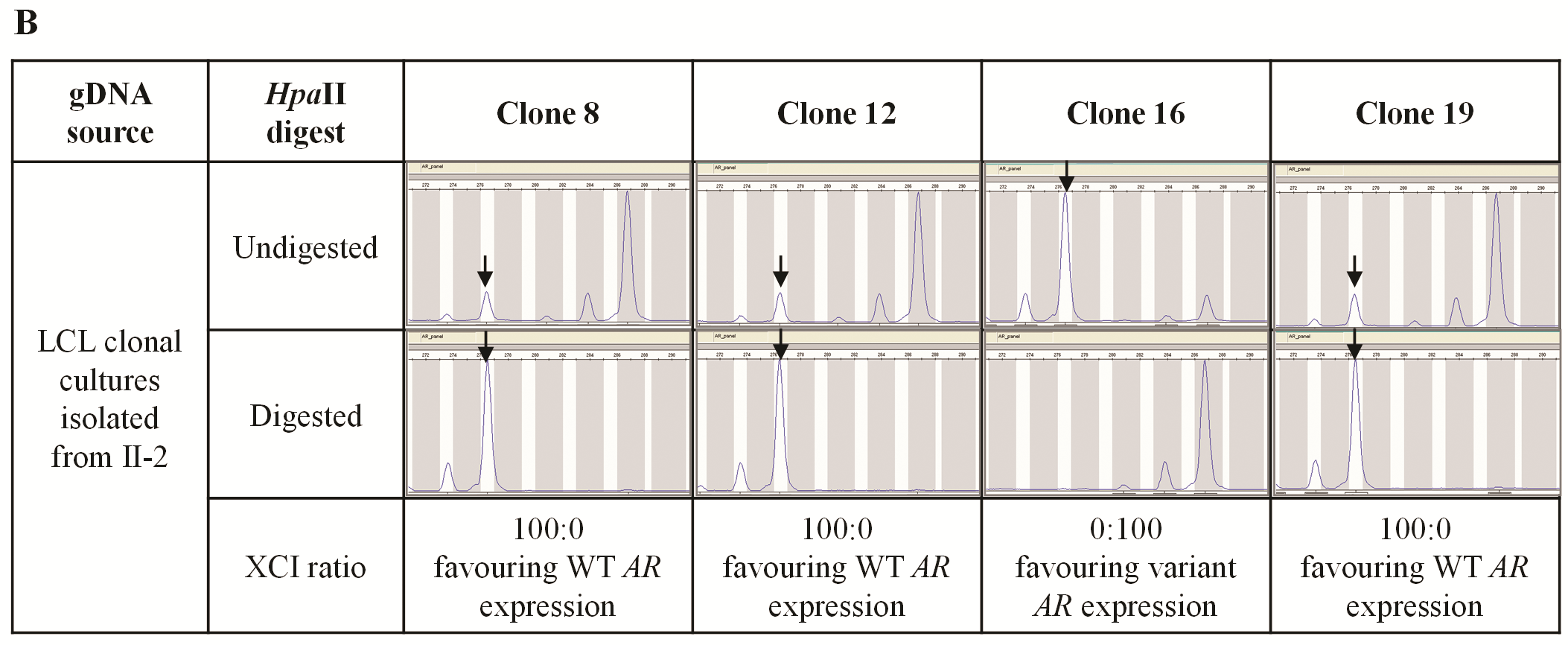


**SUPPLEMENTARY TABLES**

**Supplementary Table 1.** Rare variants identified after filtering of X-exome sequencing data of an obligate carrier female III-2. *In silico* predictions using select tools are also shown. *GPKOW* variant submitted to the Leiden Open Variation Database version 3.0 (LOVD), variant ID: 166300.

|  |  |  | *In silico* predictions | | | | |
| --- | --- | --- | --- | --- | --- | --- | --- |
| Gene | Variant (hg19) | Amino acid change | PolyPhen 2 | SIFT | CADD score: PHRED | ExAC pLI | ExAC missense constraint Z score |
| *RPGR* | chrX:g.38145178A>C | p.(Val1025Gly) | Benign | Deleterious | 8.354 | 1 | 1.48 |
|  | NG_009553.1:g.46611T>G; NM_001034853.1:c.3074T>G |  |  |  |  |  |  |
| *GPKOW* | chrX:g.48978967C>T | NA | N/A | N/A | 9.3 | 0.98 | 0.42 |
|  | NG_021310.2:g.6126G>A; NM_015698.4:c.331+5G>A |  |  |  |  |  |  |
| *MAGIX* | chrX:g.49022574A>C | p.(Thr205Pro) | Benign | Tolerated | 0.048 | 0 | 1.09 |
|  | NM_001099681.1:c.613A>C |  |  |  |  |  |  |
| *CYSLTR1* | chrX:g.77528459T>A | p.(Asn262Ile) | Benign | Tolerated | 5.051 | 0 | -1.85 |
|  | NG_012809.1:g.59629A>T; NM_001282187.1:c.785A>T |  |  |  |  |  |  |
| *IRS4* | chrX:g.107977419G>A | p.(Pro719Leu) | Probably Damaging | Deleterious | 23.4 | 0.61 | 2.73 |
|  | NG_013239.1:g.7189C>T; NM_003604.2:c.2156C>T |  |  |  |  |  |  |

**Supplementary Table 2.** Predicted effects on *GPKOW* splicing due to the c.331+5G>A variant. NNSplice = Splice Site Prediction by Neural Network (http://www.fruitfly.org/seq_tools/splice.html), HSF = Human Splicing Finder, v3.0 (http://www.umd.be/HSF3/), MES = MaxEntScan, Maximum Entropy Model (http://genes.mit.edu/burgelab/maxent/Xmaxentscan_scoreseq.html) and ESE = Exonic Splicing Enhancer Finder, v3.0, 5SS_U2_human matrix for 5' donor splice sites (http://rulai.cshl.edu/cgi-bin/tools/ESE3/esefinder.cgi).

| **Prediction Tool** | **WT Score** | **MT Score** | **Interpretation** |
| --- | --- | --- | --- |
| NNSplice | 0.98 | 0.14 | Variant site below detection threshold of 0.4 |
| HSF3.0 | 94.00 | 81.82 | WT splice site broken -12.94% |
| MaxEntScan | 9.89 | 3.31 | 6.58 times less likely to be used as a splice site compared to WT |
| ESEfinder3.0 | 10.38 | 6.94 | Variant site detected above threshold of 6.67 but score is reduced |

**Supplementary Table 3.** Summary of affected status, *IRS4*, *GPKOW* and *AR* variant genotypes, and XCI ratios for all tested family members. Height and occipitofrontal circumference (OFC) centiles are shown where available. Data shows *GPKOW* c.331+5G>A allele is in linkage disequilibrium with *AR* c.170T>A, p.(Leu57Gln) variant, and both segregate with obligate carrier and affected status. Skewing of XCI (peripheral blood DNA) does not appear to be related to either variant. Obligate carrier females are generally of short stature and OFC measurements are below average, whereas unaffected males and wild type females are of normal height and OFC, except for III-8.

| **Individual** | **Affected status** | **Carrier of *IRS4* c.2156C>T,** **p.(Pro719Leu)** | **Carrier of *GPKOW* c.331+5G>A** | **Carrier of *AR* c.170T>A, p.(Leu57Gln)** | **Peripheral Blood XCI result** | **Height centile** | **OFC centile** |
| --- | --- | --- | --- | --- | --- | --- | --- |
| I-2 | Obligate carrier female | Yes | Yes | Yes | 71% expressing WT | - | - |
| II-2 | Obligate carrier female | Yes | Yes | Yes | 25% expressing WT | 3rd | 2nd |
| II-3 | Unaffected male | No | No | No | - | 75th | 75th |
| II-4 | Unaffected male | Yes | No | No | - | 75th | 75th |
| II-8 | Obligate carrier female | No | Yes | Yes | 94% expressing WT | <1st | 2nd |
| III-2 | Obligate carrier female | Yes | Yes | Yes | 99% expressing WT | 50th | 2nd-10th |
| III-4 | Wild type female | No | No | No | 97% | 25th | 25th |
| III-6 | Unaffected male | No | No | No | - | - | - |
| III-8 | Wild type female | No | No | No | 62% | 25th | 2nd |
| IV-1 | Affected male | Yes | Yes | Yes | - | - | - |

**Supplementary Table 4.** Primer sequences

| **Primer** | **Sequence 5’→3’** |
| --- | --- |
| GPKOW g1F | CCCACCACGTTTGAAAGAGC |
| GPKOW g1R | CACCGCAGAGATTTCCAAGG |
| GPKOW g2F | ACTGACCCTGCCATTCTCC |
| GPKOW g2R | GACAGGGCAAAGAAAGGCTG |
| GPKOW g3F | CCCAGTGTAAGCAAAGCCTG |
| GPKOW g3R | GGGATCTTGATTGCAGCCTG |
| GPKOW g4F | TTTGGGCAGGAAGAGGAAGT |
| GPKOW g4R | ATTCCTCCATCTGACCCTCG |
| GPKOW g5F | CCACAACCCCTACCTGACTT |
| GPKOW g5R | GCCAACTCCCCTAGCTCTAC |
| GPKOW g6F | CTTGAACAGGCCAGATGAGC |
| GPKOW g6R | CCTTCTATGAGCCCGCCTT |
| GPKOW g7F | GGCAACTGGAAAGTGCATCT |
| GPKOW g7R | CTTGATCAACCTCCAGCTGC |
| GPKOW g8F | ACTCCCCTCTCCCTTATCCA |
| GPKOW g8R | ACAATGATCTTCCCACCTTCTG |
| GPKOW g9F | GTAGCAGGTCTCCCAGTGG |
| GPKOW g9R | TAGGTGGTTCTGGTTTGGCA |
| GPKOW g10F | CTCCCAACTTAGCCTCCCAA |
| GPKOW g10R | TGTCCTGTACCCTGAGCAGA |
| GADD45B-F | CAGCTACTGCGAAGAAAGC |
| GADD45B-R | GTTTGTGGCAGCAACTCAAC |
| HPRT-F | TGACACTGGCAAAACAATGCA |
| HPRT-R | GGTCCTTTTCACCAGCAAGCT |
| GPKOW-Exon 1-F | ACTGCCCCAATTTCATTCGG |
| GPKOW-Exon 5-R | CTTGGGCCTCAGTGAGTTGA |
| AR-F | TCCAGAATCTGTTCCAGAGCGTGC |
| AR-R | FAM-GCTGTGAAGGTTGCTGTTCCTCAT |
| FRAXA-F | GCTCAGCTCCGTTTCGGTTTCACTTCCGGT |
| FRAXA-R | HEX-AGCCCCGCACTTCCACCACCAGCTCCTCCA |
| GPKOW exon 2-3F | GCTCATTGCGGAATCCAAGAAG |
| GPKOW exon 3-4R | CTCCTCTGGCACTGTCTCTG |
| ACTB_RT_F | ATGGGTCAGAAGGATTCCTATGTG |
| ACTB_RT_R | TGTTGAAGGTCTCAAACATGATCTGG |
| IRS4_g1F | TGACAGAGGAGCCACGAAAG |
| IRS4_g1R | CTGTCCCAAAGGTGAGCTCC |

**References**

1. Hu H, Haas SA, Chelly J *et al*: X-exome sequencing of 405 unresolved families identifies seven novel intellectual disability genes. *Mol Psychiatry* 2016; **21:** 133-148.

2. Lek M, Karczewski KJ, Minikel EV et al: Analysis of protein-coding genetic

variation in 60,706 humans. *Nature* 2016; **536**: 285-291.

3. Neitzel H: A routine method for the establishment of permanent growing lymphoblastoid cell lines. *Hum Genet* 1986; **73:** 320-326.

4. Crawford J, Lower KM, Hennekam RC *et al*: Mutation screening in Borjeson-Forssman-Lehmann syndrome: identification of a novel de novo PHF6 mutation in a female patient. *J Med Genet* 2006; **43:** 238-243.

5. Thouin MM, Giron JM, Hoffman EP: Detection of nonrandom X chromosome inactivation. *Current protocols in human genetics* 2003; **Chapter 9:** Unit 9 7.
